# Impact of Early Limitation of Therapeutic Effort in Elderly COVID-19 Patients Admitted to the Intensive Care Unit—A Cohort Study

**DOI:** 10.3390/jpm12091501

**Published:** 2022-09-14

**Authors:** Thomas Lacoste-Palasset, Laetitia Sutterlin, Aymen M’Rad, Louis Modestin, Vianney Mourman, Adrien Pepin-Lehalleur, Isabelle Malissin, Giulia Naim, Caroline Grant, Emmanuelle Guérin, Jean-Michel Ekhérian, Nicolas Deye, Bruno Mégarbane, Sebastian Voicu

**Affiliations:** 1 Department of Medical and Toxicological Critical Care, Lariboisière Hospital, APHP, 75010 Paris, France; 2 INSERM UMRS-1144, Paris Cité University, 75006 Paris, France; 3 Mobile Palliative Care Team, Lariboisière Hospital, APHP, 75010 Paris, France; 4 INSERM UMRS-942, MASCOT, 75010 Paris, France

**Keywords:** COVID-19, death, elderly, frailty, intensive care unit, limitation of therapeutic effort

## Abstract

(1) Background: Admission to the ICU and intensity of care provided to elderly COVID-19 patients are difficult choices guided by the expected patient-centered benefits. However, the impact of an early discussion of limitation of therapeutic effort (LTE) has been poorly investigated. (2) Methods: We performed a single-center retrospective cohort study including all ≥70-year-old COVID-19 patients admitted to the ICU. Factors associated with early LTE discussion (defined as before or up to 2 days post-ICU admission) and in-hospital mortality were evaluated. (3) Results: Eighty-two patients (59 M/23 F; 78 years (74–82) [median (interquartile range)]; 43/82 with LTE) were included. The in-hospital mortality rate was 55%. Early LTE was decided upon for 22/82 patients (27%), more frequently in older (*p* < 0.001) and frailer patients (*p* = 0.004). Using a multivariable logistic regression model including clinical frailty scale grade ≥4, hospital acquisition of COVID-19, ventilation support modality and SOFA score on admission, early LTE was not associated with mortality (adjusted odds ratio = 0.57 (0.15–2.00), *p* = 0.39). LTE resulted in less frequent invasive mechanical ventilation (23% versus 65%, *p* = 0.001), renal replacement therapy (5% versus 27%, *p* = 0.03) and norepinephrine infusion (23% versus 60%, *p* = 0.005), and shorter ICU stay (6 days (2–12) versus 14 days (7–24), *p* = 0.001). (4) Conclusions: In this small sample exploratory study, we were unable to demonstrate any increase in in-hospital mortality associated with early LTE discussion in elderly COVID-19 patients while reducing the use of organ support techniques. These findings require confirmation in larger studies.

## 1. Introduction

Admission to the intensive care unit (ICU) represents an ethical dilemma in the elderly [1]. Deciding in which patients invasive and advanced life support measures should be initiated or not requires extensive experience and in-depth discussion between the patient, their next of kin and the caregivers in charge in order to better delineate goals of care. Identification of early predictive factors of death and expected case-by-case ICU benefits is recommended before ICU admission. However, even after ICU admission, implementation of invasive organ support techniques can still be discussed among caregivers at any time and subsequently not initiated [2]. Despite remarkable regional variability, end-of-life care practices in the ICU during the last decade showed increasing prevalence of limitations in life-prolonging therapies and decreasing proportion of deaths without therapeutic limitations [3,4]. However, the ideal timing to discuss limitation of therapeutic efforts (LTE) is unknown and has been poorly investigated. Some caregivers might consider LTE and the subsequent withholding or withdrawing of therapeutics as a loss of chance whereas others as a barrier against unreasonable obstinacy.

Since the coronavirus disease 2019 (COVID-19) pandemic, these issues have become more concerning given the limitations in ICU bed availability and severity of pulmonary injuries with expected catastrophic outcomes in the elderly [5,6]. In most life-threatening COVID-19 presentations, optimal management may require mechanical ventilation, improvement may take time, and survival, evaluated at 28% 6 months post-ICU admission in patients aged 80 years or older [7], may be poor. Due to the expected prolonged clinical course (e.g., an average of 12–14 day mechanical ventilation and >1 month ICU stay) [8,9], ICU admission and level of care intensity in elderly and/or frail patients need to be weighed against expected benefits. Different prognosticators in elderly COVID-19 patients have been identified based on large multicenter studies [10,11]; however, the impact of LTE is still not fully understood due to wide variations in practice between countries and ICUs [12,13,14]. Therefore, we designed this study aiming to investigate the impact of early LTE discussion in critically ill COVID-19 elderly patients on (i) in-hospital mortality; (ii) the number of days alive out of hospital at day 180 after ICU admission; and (iii) the therapeutic burden.

## 2. Materials and Methods

### 2.1. Study Design

We conducted a 1-year single-center retrospective observational cohort study including all successive patients aged ≥70 years admitted to our ICU with COVID-19-related respiratory distress from 1 March 2020 to 17 March 2021 (i.e., from the beginning of the first wave until the end of the third wave). Diagnosis of severe acute respiratory syndrome coronavirus-2 (SARS-CoV-2) infection relied on positive polymerase chain reaction (PCR, Cobas^®^ SARS-CoV-2 Test, Roche, France; sensitivity limit, 40 cycles) using nasopharyngeal swabs or upper respiratory/bronchial samples. Pulmonary involvement was assessed based on history, physical examination, hypoxemia requiring >6 L/min oxygen and thoracic computed tomography scan if available. Patients aged less than 70 years, patients with negative SARS-CoV-2 PCR and COVID-19 patients admitted with emergencies unrelated to respiratory failure were not included.

This study was performed in agreement with the 2013 Declaration of Helsinki of the World Medical Association. It was part of the COVID-ICU and French COVID-19 cohort registries and received approval from the ethics committee of our institution (N°, CE-SRLF-20-23). In accordance with the ethical standards of French legislation, informed consent was waived due to the non-interventional study design, which did not modify existing diagnostic or therapeutic strategies. Only the non-opposition of the patient or their legal representative was collected and subsequently, if an opposition was formulated, the patient was not included in the study. Data were collected from patients’ records in an anonymized database. Access to the identifiable data was only granted to the hospital personnel involved in the patient management. No data used in the study were re-identifiable.

### 2.2. Patient Management and LTE Discussion

Diagnosis and severity of acute respiratory distress syndrome (ARDS) relied on the Berlin definition [15]. Supportive care included high-flow nasal oxygen (HFNO), optimized non-invasive or invasive mechanical ventilation, sedation and muscular paralysis according to guidelines [16] as well as norepinephrine to maintain mean arterial pressure to at least 65 mmHg. Prone positioning was initiated in patients with PaO_2_/FiO_2_ ratio < 150 mmHg and terminated based on the PROSEVA study criteria [17]. Nitric oxide was initiated in case of refractory hypoxemia not responding to prone positioning as rescue therapy, with or without almitrine infusion.

COVID-19 patients hospitalized during the first wave (March–May 2020) received dexamethasone 20 mg for five days followed by 10 mg for five additional days as standard care in ARDS patients [18]. Since June 2020, dexamethasone 6 mg/day for 10 days according to the RECOVERY study [19], has been systematically implemented. In the absence of patient improvement, the corticosteroid dose regimen was reinforced using Villar’s protocol [18], with a second course in the most severe cases with persisting deterioration. Additional immunomodulatory therapies including hydroxychloroquine, azithromycin, anti-interleukin-6-receptor (tocilizumab) and anti-interleukin-1-receptor (anakinra) were administered, based on individual inflammatory parameters, comorbidities and response to supportive care. 

If required, LTE discussion took place in accordance with the French law in the presence of doctors and caregivers in charge of the patient and a doctor from outside the ICU able to provide an independent opinion regarding the patient status (most often, Dr V.M.) LTE discussion was not mandatory for all patients and could be repeated at different times of ICU stay if needed. Early LTE discussion was usually performed to delineate the therapeutic project and limit organ support techniques before ICU admission or during the first two days of ICU stay (day 1 and day 2 post-admission), aiming to prevent therapeutic relentlessness. By contrast, late LTE discussion was performed later during the ICU stay after a prolonged clinical course with an expected unfavorable outcome to avoid therapeutic relentlessness. The French law states that medical procedures for the purpose of prevention, diagnosis, or treatment based on the updated medical knowledge should not incur disproportionate risks compared to the expected benefits. The medical procedures should not lead to therapeutic relentlessness and, if deemed useless, disproportionate, or without another goal but to artificially maintain life, they may be discontinued or not initiated [20].

LTE discussion followed a shared decision-making model [21] and considered the patient’s advance directives, living wills and any information provided by next of kin regarding their views on implementation of organ support techniques. Age, comorbidities, frailty, disease severity, organ failure, available objective prognosticators and therapeutic options were also considered as recommended [22].

### 2.3. Study Objectives

The main objective was to evaluate the impact of early LTE discussion versus non-early LTE discussion on the risk of in-hospital mortality in elderly COVID-19 patients admitted to the ICU. Since circumstances and patient status at the time of early versus late LTE discussions are quite distinct, we decided to compare patients with early LTE versus patients without early LTE (i.e., late/no LTE) and did not consider late LTE as a separate group. The secondary objectives were to investigate the baseline differences between patients in the early versus non-early LTE group and the impact of early LTE discussion on the need for invasive mechanical ventilation, the need for renal replacement therapy, the occurrence of septic shock, the lengths of ICU and in-hospital stay, and the number of days alive out-of-hospital at day 180. The therapeutic burden was defined as an implementation of organ support (i.e., renal replacement, use of norepinephrine, and/or invasive mechanical ventilation) in patients who died in hospital despite such supports.

### 2.4. Data Collection and Parameter Definitions

The main demographic, clinical, biological, therapeutic and outcome data were collected retrospectively using medical records and the last date of follow-up was defined as 180 days after ICU admission. The date of the first symptoms and dates of the hospital and ICU admissions were also recorded. Extension of ground glass opacities and consolidations on chest computed tomography (CT) scan at the nearest time of ICU admission was graded visually by a senior pulmonologist (T.L.-P.) using a 0-to-5 scale (0 indicating no; 1, less than 5%; 2, 5–25%; 3, 26–49%; 4, 50–75%; and 5, more than 75% lung involvement) [23]. The clinical frailty scale (CFS; an ordinal hierarchical scale of 9 ranks) was used to evaluate the overall fitness level or frailty on ICU admission [24]. If the patient was unable to communicate, the physician in charge obtained the information from the relatives. Patients with a CFS grade ≤3 were considered fit as previously defined [11]. The Charlson comorbidity index [25] and the Sequential Organ Failure Assessment (SOFA) score [26] were also calculated on ICU admission. Acute renal failure was classified based on the Kidney Disease Improving Global Outcome (KDIGO) criteria [27]. Norepinephrine doses were expressed in equivalents of norepinephrine-base. Life-sustaining treatment decisions were also collected. LTE discussion was defined as early if occurring before ICU admission or during the first two days post-ICU admission (i.e., day 1 or day 2) and as late if occurring >2 days after ICU admission. All patients who had an early LTE were classified in the “early LTE group” and the rest of the patients (i.e., who only had late LTE or no LTE at all) in the “non-early LTE group”, regardless of their timing of death. In-hospital mortality was defined as death occurring in the ICU or in the medical ward. In-hospital survival was defined as home discharge or secondary transfer to long-term rehabilitation care center. The number of days alive out-of-hospital was calculated starting from hospital discharge. Data were retrieved using Orbis^TM^ software (Agfa HealthCare France, Ivry-sur-Seine, France) used in most regional hospitals and subsequently confirmed by a phone call to the patient. 

### 2.5. Statistical Analysis

Categorical variables are expressed as frequency (percentage) and continuous variables as median (interquartile range, IQR). Comparisons between the different groups were performed using Fisher and Mann–Whitney tests as appropriate. An exploratory multivariable logistic regression model was used to determine parameters independently associated with in-hospital mortality. Early LTE and parameters present on admission associated with in-hospital mortality in the univariate analyses with a *p*-value < 0.10 were considered for inclusion in the multivariable model. The maximum number of variables was fixed as one variable for each 5–10 in-hospital mortality events. As CFS and age groups were shown to be collinear in a large previous study [10], only CFS was included in the multivariable model. Logit linearity was assessed visually for linear variables. Two variables (i.e., serum albumin concentration and CT-scan severity) did not respect the log linearity and were thus categorized as binary variables split based on their median value. Odds ratios (OR) and their 95% confidence intervals were calculated. Statistical analyses were performed using the software. R-3.6.1 for Windows^®^ (R Foundation for Statistical Computing, Vienna, Austria; https://www.r-project.org/ (accessed on 13 September 2022)).

## 3. Results

### 3.1. Patient Description

**Figure 1:**
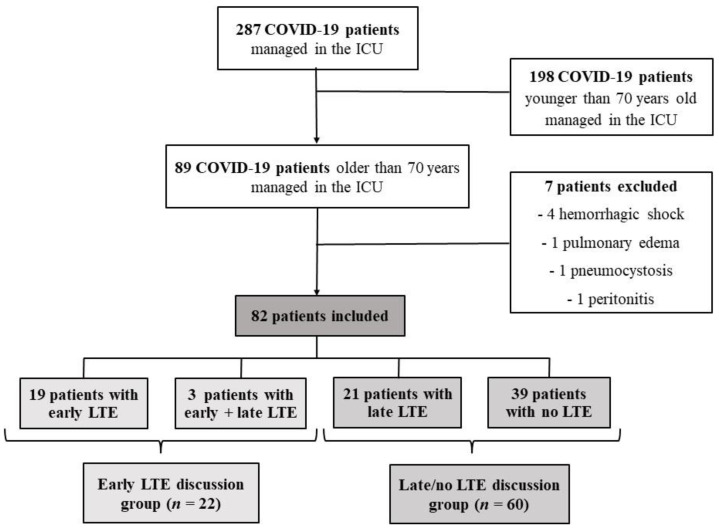
Study flowchart. COVID-19, coronavirus infectious disease-2019; ICU, intensive care unit.

During the study period, eighty-nine critically ill COVID-19 patients aged 70 years or older were admitted to our ICU (among 287 COVID-19 patients; Figure 1). Seven patients were not included as admitted for a condition other than SARS-CoV-2-attributed pneumonia including hemorrhagic shock (*n* = 4), cardiogenic pulmonary edema (*n* = 1), pneumocystosis (n = 1) and peritonitis (*n* = 1). Thus, eighty-two patients (59 M/23 F; age, 78 years (74–82); CFS grade ≤ 3, *n* = 56 (68%)) were included (Table 1).

Supportive care included mechanical ventilation (*n* = 44/82, 54%), prone positioning (*n* = 32/44, 73% of ventilated patients), nitric oxide (*n* = 13/44, 30% of ventilated patients), almitrine infusion (*n* = 3/44, 7% of ventilated patients) and venovenous extracorporeal membrane oxygenation (*n* = 1/44, 2% of ventilated patients) (Table 2). Dexamethasone was administered to 68/82 patients (83%) at 20 mg for 5 days followed by 10 mg for 5 days (*n* = 14/68, 17%), at 6 mg/day for 10 days (*n* = 40/68, 66%), or combining both regimens successively (*n* = 14/68, 17%). Tocilizumab was administered to 8/82 patients (10%). Complications included acute kidney injury (*n* = 52/82, 63%) and septic shock (*n* = 41/82, 50%). Forty-four patients (54%) died in the ICU and one additional patient died in the hospital after ICU discharge. Median length of ICU stay was 12 days (6–21) and median length of hospital stay was 17 days (10–34). Among the 37 hospital survivors, 24/37 (65%) returned home directly after hospital discharge and 13/37 (35%) were sent to a rehabilitation care center. Median number of days alive out-of-hospital at day 180 was 0 days (0–136) overall and 143 days (119–161) among in-hospital survivors.

### 3.2. Prognostic Factors of In-Hospital Death

Based on univariate analyses, only hospital-acquired COVID-19 (OR = 4.12 (1.18–19.30), *p* = 0.02) and SOFA score on admission (OR = 1.28 (1.07–1.60), *p* = 0.01) were significantly associated with in-hospital death, while age ≥80 (OR = 2.27 (0.89–6.12), *p* = 0.09), CFS grade ≥4 (OR = 2.42 (0.93–6.74), *p* = 0.08) and initial ventilation support modalities (non-invasive ventilation versus HFNO and conventional oxygen therapy, OR = 2.43 (0.86–7.27); and invasive mechanical ventilation versus HFNO and conventional oxygen therapy, OR = 4.51 (1.45–18.16); overall *p* = 0.08) were not significantly associated with in-hospital mortality (Table 3). During the ICU stay, norepinephrine infusion (OR = 9.97 (3.73–29.35), *p* < 0.001), KDIGO 3 acute kidney injury (OR = 11.29 (3.73–42.77), *p* < 0.001) and peak norepinephrine infusion rate >0.5 µg/kg/min (OR = 16.00 (2.49–316.87), *p* = 0.01) were also associated with in-hospital mortality. Of note, among the 13 patients in whom nitric oxide was initiated, none survived, while only one among the 23 septic shock patients (4%) treated with norepinephrine > 0.5 µg/kg/min survived.

### 3.3. Characteristics of Patients with Early LTE Discussion

Overall, LTE discussion was decided upon for 43/82 patients (52%). LTE was discussed early in 22/82 patients (27%) including 6/22 (27%) before ICU admission and 16/22 (73%) during the first two days post-admission. All early LTE discussions were focused on treatment withholding except one on end-of-life decision. In the non-early LTE group, 21/60 (35%) and 39/60 patients (65%) had only a late or no LTE discussion, respectively (Figure 1). Characteristics including baseline differences between patients in the early (*n* = 22) versus non-early LTE discussion group (*n* = 60) are expressed in Table 1. Early LTE patients were older (83 years (78–85) versus 77 years (73–79), *p* < 0.001) and had more often hospital-acquired COVID-19 (41% versus 10%, *p* = 0.003). They had a more elevated CFS grade (4 (3–6) versus 3 (2–3), *p* = 0.003) and tended to more often have a CFS grade ≥4 (50% vs. 25%, *p* = 0.06). 

During early LTE discussion, it was decided not to perform invasive mechanical ventilation (*n* = 17/22, 77%, including one patient intubated in the prehospital setting), cardiac arrest resuscitation (*n* = 21/22, 96%), norepinephrine infusion (*n* = 15/22, 68%) or renal replacement therapy (*n* = 15/22, 68%, including one patient on chronic dialysis). One patient received mechanical ventilation despite initial LTE decision not to intubate; he died 13 days later due to refractory hypoxemia.

In-hospital death occurred in 13/22 (59%) in the early LTE group versus 32/60 (53%) in the non-early LTE group (*p* = 0.80).

### 3.4. Impact of LTE Discussion on Outcomes

**Figure 2:**
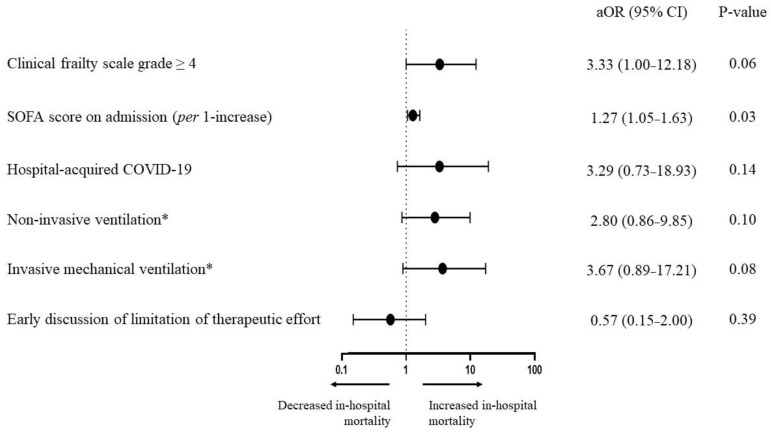
Forrest plot representing the odds ratios of in-hospital mortality according to the clinical frailty scale grade, the hospital-acquired origin of COVID-19, the ventilation support modality initiated during the first 24 h and the early discussion of limitation of therapeutic effort. Comparisons were performed using a multivariable logistic regression model. Points represent the adjusted odds ratios and lines represent 95% confidence intervals. * Compared with high-flow nasal oxygen or conventional oxygen taken as reference. aOR, adjusted odds ratio; CI, confidence interval; SOFA, Sequential Organ Failure Assessment.

Based on a univariate analysis, early LTE discussion was not associated with in-hospital mortality (OR = 1.26 (0.47–3.49), *p* = 0.64) (Table 3). Consistently, early LTE was not associated with in-hospital mortality (adjusted OR = 0.57 (0.15–2.00), *p* = 0.39) in a multivariable logistic regression model including CFS grade ≥4, hospital-acquired nature of COVID-19, initial respiratory support modality and SOFA score on admission, as shown in Figure 2. Patients with early LTE discussion less frequently received invasive mechanical ventilation (23% versus 65%, *p* = 0.001), norepinephrine infusion (23% versus 60%, *p* = 0.005) and renal replacement therapy (5% versus 27%, *p* = 0.03). They had a shorter length of ICU stay (6 days (2–12) versus 14 days (7–24), *p* = 0.001). The proportion of survivors requiring rehabilitation after ICU stay was not different between both groups (3/9 (33%) versus 10/28 (36%), *p* = 1). The number of days alive out-of-hospital at day 180 was evaluated in 79 patients with no patient lost to follow-up in the early LTE group but three patients lost to follow-up in the non-early LTE group. No difference was observed between the two groups (0 days (0–90) versus 0 days (0–139), *p* = 0.40) and among in-hospital survivors (141 days (90–155) versus 143 days (125–162), *p* = 0.34) (Table 2). The number of patients in whom a therapeutic burden (defined as organ support initiation resulting however ultimately in in-hospital death) was identified was significantly lower in the early LTE group (18% versus 48%, *p* = 0.01).

## 4. Discussion

In this cohort of critically ill COVID-19 elderly patients managed in the ICU, early LTE discussion was not associated with increased risk of in-hospital death. In addition, we found that early LTE discussion was associated with lower use of organ support techniques and shorter duration of ICU stay.

Our observed mortality rate (55%) was slightly higher than the one reported in the French nationwide COVID-ICU Network (46%) [8], possibly related to older patients (34% versus 16% patients older than 80 years). Noteworthy, in patients requiring high-dose norepinephrine and rescue nitric oxide, survival rates were extremely low (4.2% and 0%, respectively), thus questioning the utility of such support therapies in elderly COVID-19 patients.

LTE is widely used and may be considered as a quality indicator of ICU care [28]. LTE includes life support withholding and withdrawing, and from an ethical point of view, the distinction between the two remains a matter of debate [22,29]. By limiting therapeutic relentlessness, early LTE discussion is supposed to be beneficial to the patients and their next of kin. Advanced care planning on end-of-life decision in the elderly outside of ICU was shown to be associated with significantly reduced stress, anxiety and depression in the families of the patients who died [30]. Such a strategy allows patient management to be focused on care deemed appropriate and in case of ineffectiveness, to rapidly initiate palliative symptom-oriented care. Furthermore, a recent study showed that perception of inappropriate care was a major risk factor for psychological distress among healthcare providers [31]. Early LTE discussion might help in reducing psychological distress by allowing a better understanding of a well-established therapeutic plan and should thus be investigated in further large randomized studies.

Twenty-two patients (27%) were subject to early LTE discussion. Surprisingly, despite being frailer and older, they had a survival rate not different from that of non-early LTE patients, suggesting that early LTE application did not worsen the outcome. The number of days alive out-of-hospital at day 180 was also not different between the two groups. Additionally, early LTE discussion probably limited therapeutic burden, with shorter lengths of ICU and hospital stay and less frequent use of organ support techniques. In a previous large multicenter study including non-COVID-19 patients, LTE on admission, performed in older patients with more altered functional status and higher initial risk of death than patients without these limitations, was associated with increased ICU mortality [32]. By contrast, in the present exploratory study, we showed that early LTE discussion was most likely not associated with in-hospital mortality in elderly COVID-19 patients. This discrepancy was probably explained by the extremely prolonged disease course in our COVID-19 patients compared to the conditions assessed in non-COVID-19 patients (i.e., ICU length of stay of 12 days (6–21) versus 3 days (1–6) [32]), decreasing the chances of recovery after prolonged organ support. Therefore, we strongly believe that chronic frailty (i.e., pre-existing before the onset of COVID-19 symptoms), once identified in critically ill COVID-19 elderly patients, should trigger early LTE discussion, as suggested by a previous multicenter study in the non-COVID-19 elderly patients [2].

Our study has limitations. We could not rule out an underpowered analysis due to limited sample size and center-related particularities due to a single-center study design, thus, larger multicenter studies are required. Moreover, a larger study, possibly spanning the entire duration of the epidemic, may have provided more detailed insights into the effect of early LTE on patient outcome in general and within different waves. Our inclusions were stopped in March 2021 when the preliminary data analysis modified our management of elderly patients with an early LTE discussion performed on a regular basis, precluding an extended comparative study. Early LTE discussion was the exposure of interest; however, patients in the early LTE and non-early LTE groups differed especially in age and CFS grade. Although we adjusted for the effect of covariates regarding the impact of early LTE discussion on survival to hospital discharge, the lack of statistical power should be acknowledged. Otherwise, our study lacked information on patients not managed in the ICU as result of pre-ICU LTE precluding their admission. It is possible that during the overwhelming first COVID-19 wave (March–April 2020), patients aged over 70 years and, more likely, beyond 80 years were not admitted to the ICU due to lack of available resources. These data were not recorded and could not be retrieved for the present study. Finally, although we showed that early LTE was not associated with in-hospital mortality, we did not evaluate the satisfaction of caregivers and patients’ families with this procedure, which could be evaluated in further studies.

## 5. Conclusions

In this small-sample exploratory study, we were unable to demonstrate any increase in in-hospital mortality associated with early LTE discussion in the elderly COVID-19 patients, which resulted in less frequent invasive mechanical ventilation and renal replacement therapy. Early LTE discussion before ICU admission or during the first two days post-ICU admission should be encouraged, especially in case of chronic frailty. Our findings should be confirmed in future larger multicenter prospective studies.

## Figures and Tables

**Table 1:** Characteristics of 82 critically ill COVID-19 elderly patients on intensive care unit admission according to the early versus non-early discussion of limitation of therapeutic effort.

	Overall (*n* = 82)	Non-Early LTE Discussion (*n* = 60)	Early LTE Discussion (*n* = 22)	*p*-Value
Demographics				
Age, years	78 (74–82)	77 (73–79)	83 (78–85)	**<0.001**
Age > 80 years	28 (34)	13 (22)	15 (68)	**<0.001**
Male gender	59 (72.0)	43 (71.7)	16 (72.7)	1
Body mass index, kg·m^−2^	27.1 (24.0–30.6)	27.7 (24.2–31.0)	25.2 (23.4–28.9)	0.46
Clinical frailty scale	3 (2–4)	3 (2–3)	4 (3–6)	**0.004**
Clinical frailty scale ≥4	26 (32)	15 (25)	11 (50)	**0.048**
Comorbidities				
Hypertension	56 (68)	43 (72)	13 (59)	0.30
Ischemic cardiomyopathy	14 (17)	10 (17)	4 (18)	1
Chronic heart failure	11 (13)	6 (10)	5 (23)	0.16
Chronic kidney disease	15 (18)	13 (22)	2 (9)	0.33
Peripheral arterial disease	5 (6)	2 (3)	3 (14)	0.12
Cerebrovascular disease	6 (7)	5 (8)	1 (5)	1
COPD	7 (9)	5 (8)	2 (9)	1
Diabetes mellitus	31 (38)	27 (45)	4 (18)	**0.04**
End organ damage	18 (22)	16 (59)	2 (50)	1
Connective tissue disease	2 (2)	1 (2)	1 (5)	0.47
Gastro-intestinal peptic ulcer	2 (2)	2 (3)	0 (0)	1
Cancer	10 (12)	5 (8)	5 (23)	0.12
Metastasis	2 (8)	1 (2)	1 (5)	1
Atrial fibrillation	11 (13)	5 (8.3)	6 (27)	0.06
Charlson comorbidity index	5 (4–6)	4 (4–6)	6 (4–6)	**0.03**
Charlson index null besides age	24 (29)	17 (28)	7 (32)	0.79
Disease history and severity on ICU admission				
Hospitalization for COVID-19	67 (82)	54 (90)	13 (59)	**0.003**
Symptom onset to ICU, days	8 (6–12)	8 (6–12)	9 (7–13)	0.39
Hospital to ICU admission, days	2 (0–4)	2 (0–4)	2 (0–6)	0.68
Hospital-acquired COVID-19	15 (18)	6 (10)	9 (41)	**0.003**
Hospital to ICU admission, days	20 (17–45)	21 (19–37)	20 (15–48)	0.91
Plasma D-dimer, mg/L	1.58 (0.97–3.14)	1.91 (1.10–3.48)	1.28 (0.70–2.26)	0.07
Serum albumin, g/L	26.3 (23.1–28.5)	25.7 (23.0, 27.7)	28.0 (25.8, 28.8)	0.09
CT-scan severity category	4 (3–4)	4 (3–4)	4 (3–4)	0.88
CT-scan severity (categories 4 to 5)	37/57 (65)	29/44 (66)	8/13 (62)	0.75
SOFA score	4 (3–6)	4 (3–6)	4 (4–5)	0.55
Admission during first wave	26 (32)	18 (30)	8 (36)	0.60
Respiratory support during the first hours				
Oxygen supply modalities				**0.05**
HFNO/conventional oxygen	43 (52)	33 (55)	10 (46)	
Non-invasive ventilation	22 (27)	12 (20)	10 (46)	
Invasive ventilation	17 (21)	15 (25)	2 (9)	
PaO_2_/FiO_2_ ratio, mmHg	114 (80–181)	119 (91–192)	100 (61–140)	0.08

Data are presented as median (interquartile range) or *n* (percentage). Statistically significant *p*-values are indicated in bold. COPD, chronic obstructive pulmonary disease; COVID-19, coronavirus disease-2019; CT, computed tomography; FiO_2_, inspired fraction of dioxygen; HFNO, high-flow nasal oxygen; ICU, intensive care unit; LTE, limitation of therapeutic effort; PaO_2_, Partial pressure of dioxygen in arterial blood; SOFA, Sequential Organ Failure Assessment.

**Table 2:** Management and outcome in 82 critically ill COVID-19 elderly patients in relation to early versus non-early discussion of limitation of therapeutic efforts.

	Overall	Non-Early LTE Discussion	Early LTE Discussion	*p-*Value
	(*n* = 82)	(*n* = 60)	(*n* = 22)	
Ventilation management				
Non-invasive ventilation	36 (44)	23 (38)	13 (59)	0.13
Invasive mechanical ventilation	44 (54)	39 (65)	5 (23)	**0.001**
Duration, days	15.5 (9.0–25.8)	16.0 (9.0–28.0)	11.0 (11.0–13.0)	0.33
Prone positioning	32/44 (73)	29/39 (74)	3/5 (60)	0.60
Nitric oxide	13/44 (30)	11/39 (28)	2/5 (40)	0.62
Almitrine infusion	3/44 (7)	2/39 (6)	1/5 (9)	1
ECMO	1 (1)	1 (2)	0 (0)	1
Anti-COVID-19 therapies				
Dexamethasone	68 (83)	51 (85)	17 (77)	0.51
6 mg/day dose regimen	54 (79)	40 (78)	14 (82)	1
20 mg/day dose regimen	28 (41)	21 (41)	7 (41)	1
Tocilizumab	8 (10)	4 (7)	4 (18)	0.20
Critical care complications and outcome				
Acute kidney injury	52 (63)	40 (67)	12 (55)	0.44
KDIGO-3	30 (37)	25 (42)	5 (23)	0.13
Renal replacement therapy	17 (21)	16 (27)	1 (5)	**0.03**
Norepinephrine infusion	41 (50)	36 (60)	5 (23)	**0.005**
Norepinephrine > 0.5 µg/kg/min	23 (55)	21 (58)	2 (33)	0.38
Alive out of ICU	38 (46)	28 (47)	10 (46)	1
ICU length of stay, days	12 (6–21)	14 (7–24)	6 (2–12)	**0.001**
Alive out of hospital	37 (45)	28 (47)	9 (41)	0.80
Hospital length of stay, days	17 (10–34)	20 (14–37)	8 (4–20)	**0.001**
Need for rehabilitation in survivors	13 (35)	10 (36)	3 (33)	1
Number of days alive out-of-hospital at day 180	0 (0–136) [*n* = 79]	0 (0–139) [*n* = 57]	0 (0–90) [*n* = 22]	0.40
Number of days alive out-of-hospital at day 180 among in-hospital survivors	143 (119–161)	143 (125–162)	141 (90–155)	0.34
Therapeutic burden	34 (42)	29 (48)	4 (18)	**0.01**

Data are presented as median (interquartile range) or *n* (percentage). Statistically significant *p*-values are indicated in bold. COVID-19, coronavirus disease-2019; ECMO, extracorporeal membrane oxygenation; HFNO, high-flow nasal oxygen; ICU, intensive care unit; KDIGO, kidney disease: improving global outcome; LTE, limitation of therapeutic effort.

**Table 3:** Predictive factors of in-hospital mortality in critically ill COVID-19 elderly patients managed in the intensive care unit based on univariate analyses.

	Survivors (*n* = 37)	Non-Survivors (*n* = 45)	OR (95% CI)	*p-*Value
Demographics				
Age, (per 1-increase)	77 (74–80)	78 (74–83)	1.05 (0.97–1.15)	0.24
Age, >80 years	9 (24)	19 (42)	2.27 (0.89–6.12)	0.09
Male gender	26 (70)	33 (73)	1.16 (0.44–3.07)	0.76
CFS (per 1-increase)	3 (2–3)	3 (2–4)	1.19 (0.89–1.64)	0.25
CFS grade ≥ 4	8 (22)	18 (40)	2.42 (0.93–6.74)	0.08
Charlson null besides age	11 (30)	13 (29)	0.96 (0.37–2.53)	0.93
Disease history and severity on admission				
Hospital-acquired COVID-19	3 (8)	12 (27)	4.12 (1.18–19.30)	**0.04**
Serum albumin < 26.3 g/L	17 (57)	16 (43)	0.58 (0.22–1.53)	0.27
Plasma D-dimer, mg/L	1.99 (0.99–3.30)	1.47 (0.98–2.89)	0.99 (0.90–1.10)	0.86
SOFA score (per 1-increase)	3.0 (3.0–4.0)	4.0 (4.0–7.0)	1.28 (1.07, 1.60)	**0.01**
CT scan extension > 50%	17/25 (68)	20/32 (63)	0.78 (0.25–2.35)	0.66
Admission during first wave	12 (32)	14 (31)	0.91 (0.49, 1.71)	0.77
Respiratory support during the first hours				
Oxygen supply modality				0.08
HFNO/conventional oxygen	25 (68)	18 (40)	1	
Non-invasive ventilation	8 (22)	14 (31)	2.43 (0.86–7.27)	
Mechanical ventilation	4 (11)	13 (29)	4.51 (1.45–18.16)	
PaO_2_/FiO_2_ ratio *, mmHg	143 (82–192)	106 (72–163)	1.03 (0.64–1.69)	0.91
Management and complications in the ICU				
Dexamethasone	30 (81)	38 (85)	1.27 (0.39–4.09)	0.69
Invasive mechanical ventilation	12 (32)	32 (71)	5.13 (2.05–13.61)	**<0.001**
Prone positioning	5/12 (42)	27/32 (84)	7.56 (1.77–36.65)	**0.01**
Nitric oxide	0/12	13/32 (41)	NA **	**0.009**
Acute kidney injury KDIGO-3	4 (11)	26 (58)	11.29 (3.73–42.77)	**<0.001**
Renal replacement therapy	2 (5)	15 (33)	8.75 (2.23–58.41)	**0.006**
Norepinephrine infusion	8 (22)	33 (73)	9.97 (3.73–29.35)	**<0.001**
Norepinephrine > 0.5 µg/kg/min	1/8 (11)	22/33 (67)	16.00 (2.49–316.87)	**0.01**
Limitation of therapeutic effort				
Early LTE	9 (24)	13 (29)	1.26 (0.47–3.49)	0.64

Data are presented as median (interquartile range) or *n* (percentage). Statistically significant *p*-values are indicated in bold. CI, confidence interval; CFS, clinical frailty scale; COVID-19, coronavirus disease-2019; DXM, dexamethasone; FiO_2_, inspired fraction of dioxygen; HFNO, High-flow nasal oxygen; HR, hazard ratio; ICU, intensive care unit; KDIGO, kidney disease: improving global outcome; LTE, limitation of therapeutic effort; NA, not available; PaO_2_, Partial pressure of dioxygen in arterial blood; SOFA, sepsis-related organ failure assessment. * OR are expressed per 100 mmHg of variation. ** Since OR could not be calculated, *p* was calculated using a Fisher exact test.

## Data Availability

Bruno Mégarbane has full access to all data and takes responsibility for the data integrity and their analysis accuracy. Data supporting reported results can be obtained from the corresponding author if reasonably justified.
